# The Book World of Medicine and Science

**Published:** 1907-09-21

**Authors:** 


### The Book World of Medicine and Science.

### ETHICS AND DIETETICS.

We have received for review a food chart prepared by the well-known philanthropist, Mr. Seebohm Rowntree, of York. The chart is published by the York Health Housing Association, 3a Bootham, York, and issued at cost price—namely, 4s. per hundred, or £1 19s. per thousand. As far as we are aware this is the first attempt in this country to popularise the science of dietetics, for these charts are intended to be issued to the working classes, and indeed to find a place among the mural decoration of the artizan's home. When we remember the main object of the chart before us is ethical and not scientific, we must congratulate Mr. Rowntree and his collaborator, Mr. Davies, upon the result of their cogitations. We have on the shelves of our library many publications of the Librairie Armand Colin of a similar kind, some of them indeed published under the auspices of the French Government; and we would in this connection draw special attention to the Tableau Mural d'Hygiène. The difference, however, between Mr. Rowntree's charts and these publications is, however, from the point of view of price, a very marked one, and when we consider that the chart before us can be bought for a halfpenny, we can form an idea of its sphere of usefulness.

Much as we regret it, we are bound to say that Mr. Rowntree's science is not as good as his ethics. Speaking generally, the chart is too complicated and too much stress is laid upon the so-called calorimetric value of food. The human body is not a bomb calorimeter, and because certain foods, under certain specific conditions, develop certain empirically reckoned energy units, it does not follow that the same will happen *in vivo*. Appetite, flavour, appearance, and smell of food are physiological factors which in many cases dictate absolutely whether a given kind of food can be digested and absorbed and thus be of energy value or not. When dealing with the amount of water in food, we are upon much surer ground, and we think if a popular standard for nutritive value were required a simple, fairly accurate and efficacious one would be water content. We regret that Mr. Rowntree has made no mention of tinned, preserved, or dried foods, with the single exception of peas. There are many excellent dried milks on the market which are very cheap, and many excellent brands of preserved meat and fish. We think also nuts should have been mentioned. Our remarks must conclude with noticing the question of alcohol and alcoholic beverages, and we are sorry again to disagree with the compiler of the chart upon matters of fact. Whatever alcohol may be ethically, physiologically, when taken in certain quantities, it is in the truest and completest sense of the term a food, and we think it is a mistake to include in a popular chart *ex cathedra* statements upon subjects which are at any rate distinctly *sub judice*. We do not, as a rule, take beverages for the purpose of nutrition, and we think it would only have been fair if, above or below the diagram giving the energy value of alcoholic beverages, we had been provided with one giving that of tea.

We should not like our criticisms to discourage those responsible for the chart under consideration, and our excuse for them must be that as a scientific paper we are scientific before we are ethical. We have no doubt whatever that Mr. Rowntree's chart will encourage dietetic thrift amongst a class apt to be thriftless.

### THE DISEASE OF THE AGE.*

There is no particular reason why Dr. Saleeby should have styled worry the disease of this age—an age which, he hastens to inform us, he believes to be the greatest in human history hitherto. "Care killed the cat" in the time of Alfred the Great, and the Khoi-Khoi have a fable that the milky way is the remains of a woman who worried too much. St. Francis rebuked Brother Leo for "caring for the need of the day," but the saint condensed his sermon into a line. Dr. Saleeby needs three hundred odd pages to drive his lesson home, and when we have finished his book, interesting as it is, we are left to wonder whether he has done half as much as the Assisian to comfort us. A few points set aside for the moment, we are entirely with the author on his main arguments and conclusions. Worry is futile; it is a factor in the causation of mental disease; its cure must of necessity be psychical—these are commonplaces, truisms with which we need not quarrel. At once, therefore, we may dismiss the first twelve chapters of this work. They tell us, very clearly and at times even prettily, in the author's best "popular" style, how drink, drugs, domestic worry, "blackguardly advertisers," boredom, and religious worry are ruining us daily. We knew all that, though some of us, possibly, may not have felt it so much before we read and reflected over Dr. Saleeby's pages. It is the latter half of the book that makes the better reading, for in it the author attempts to grapple with the question, "How is this disease to be combated?" The last few chapters are thoughtful contributions to a subject which must interest all thinking men. Being such, they must of necessity give rise to some degree of controversy. Dr. Saleeby, who has obviously fallen under the spell of Professor Hoffding, gives us nothing definite. He deprecates doubt, condemns what is vulgarly called patriotism, and attempts to argue that so long as men vary religious conformity is "as impossible as it is undesirable." All three are debatable points, and the author will be among the first, we presume, to agree that there is a great deal of truth underlying Dante's line, "Che, non men che saver, dubbiar m'aggrata." This is not the place fully to discuss the metaphysical conundrums which face us when we have read Dr. Saleeby's book. Suffice it to say that they are interesting, as such things must always be, not only to the student, but to the general reader. Ours is a light age and busy, and a book that "gives to think," be it in a degree ever so little, is worth reading and study. "Worry" is not only clearly and simply written, but it is stocked with informative material, and it traverses points which no one, be he physician or layman, can reasonably ignore. As such it is to be welcomed.

* "Worry: The Disease of the Age." By C. W. Saleeby, M.D. Cassell and Co., Ltd. 6s.

